# New Era of Endoscopic Ultrasound-Guided Tissue Acquisition: Next-Generation Sequencing by Endoscopic Ultrasound-Guided Sampling for Pancreatic Cancer

**DOI:** 10.3390/jcm8081173

**Published:** 2019-08-05

**Authors:** Hiroshi Imaoka, Mitsuhito Sasaki, Yusuke Hashimoto, Kazuo Watanabe, Masafumi Ikeda

**Affiliations:** Department of Hepatobiliary and Pancreatic Oncology, National Cancer Center Hospital East, 6-5-1, Kashiwanoha, Kashiwa, Chiba 277-8577, Japan

**Keywords:** next-generation sequencing, genome sequencing, endoscopic ultrasound-guided tissue acquisition, endoscopic ultrasound-guided fine needle aspiration, endoscopic ultrasound-guided fine needle biopsy, pancreatic cancer, biomarker, precision medicine

## Abstract

Pancreatic cancer is a lethal cancer with an increasing incidence. Despite improvements in chemotherapy, patients with pancreatic cancer continue to face poor prognoses. Endoscopic ultrasound-guided tissue acquisition (EUS-TA) is the primary method for obtaining tissue samples of pancreatic cancer. Due to advancements in next-generation sequencing (NGS) technologies, multiple parallel sequencing can be applied to EUS-TA samples. Genomic biomarkers for therapeutic stratification in pancreatic cancer are still lacking, however, NGS can unveil potential predictive genomic biomarkers of treatment response. Thus, the importance of NGS using EUS-TA samples is becoming recognized. In this review, we discuss the recent advances in EUS-TA application for NGS of pancreatic cancer.

## 1. Introduction

Pancreatic cancer (PC) is one of the deadliest cancers and the fourth leading cause of cancer death. It is estimated that in 2019 approximately 45,750 patients in the United States will die of this disease [1]. Although newer combination chemotherapeutic regimens have been shown to prolong survival in patients with PC [2,3,4,5], their prognosis remains poor, with fewer than 10% of patients surviving five years after the initial diagnosis [6].

One possible reason for the poor prognosis is the lack of predictive biomarkers for therapeutic stratification, such as *EGFR* in lung cancer [7] and *BRCA1/2* in ovarian cancer [8], are still lacking in PC. Presently, a new clinical trial design called “basket trial”, for which eligibility is based on the presence of specific genomic biomarkers regardless of the involved organs, has emerged [9,10]. However, it is estimated that less than 10% of registered drug intervention trials for PC have included a molecular/biomarker stratification strategy [11]. These situations could delay the introduction of potentially effective therapies into clinical practice. 

Recently, pembrolizumab was introduced as the first major advance toward a more individualized approach for treating PC [12]. In the KEYNOTE-158 trial, pembrolizumab showed both efficacy and safety in different tumor types with microsatellite instability-high (MSI-H), and the US Food and Drug Administration approved pembrolizumab for the treatment of refractory PC with MSI-H. The importance of next-generation sequencing for both the development of new treatments and precision medicine is garnering attention. Endoscopic ultrasound-guided tissue acquisition (EUS-TA) is the primary method to obtain tissue samples from PC easily and safely. Next-generation sequencing (NGS) enables the sequencing of multiple genes in a limited number of samples obtained by EUS-TA, and allows potential mutations as therapeutic targets to be identified. In this review, we discuss the recent advances in the application of EUS-TA to NGS for PC.

## 2. Endoscopic Ultrasound-Guided Tissue Acquisition (EUS-TA)

**Figure 1:**
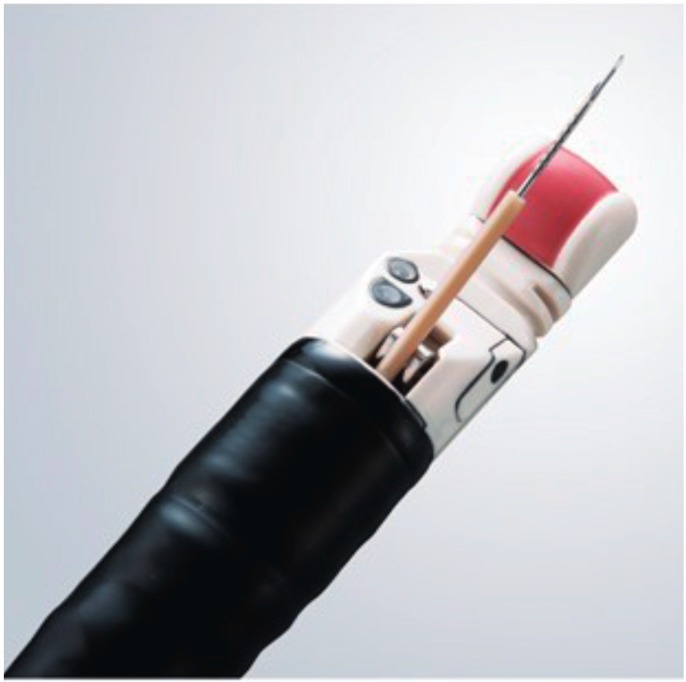
Linear echoendoscope (GF-UE160, Olympus Medical Systems, Tokyo, Japan).

**Figure 2:**
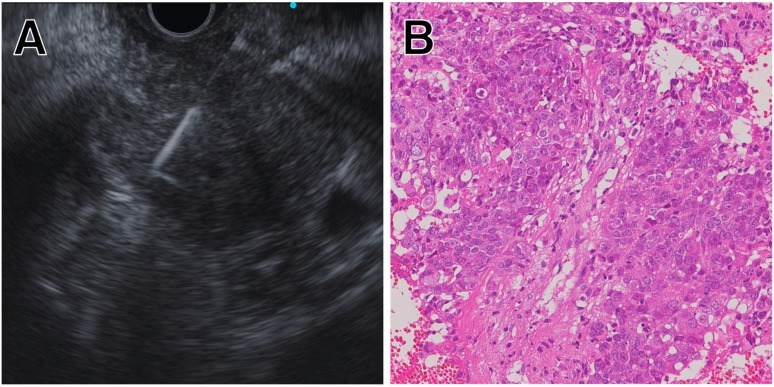
Representative case of Endoscopic Ultrasound-Guided Tissue Acquisition (EUS-TA) for pancreatic cancer (PC). (**A**) Endoscopic ultrasound-guided fine needle biopsy (EUS-FNB) of PC located in the body of the pancreas. (**B**) Tissue sample obtained by EUS-FNB showing large tissue fragments of poorly differentiated adenocarcinoma.

Endoscopic ultrasound (EUS) was designed in the early 1980s in an attempt to improve ultrasonography imaging of the pancreatobiliary system [13]. Its unique design, an ultrasonography probe attached to the tip of endoscope, allows for improved visualization of the gastrointestinal wall and its surrounding structures. In the early 1990s, the linear-array echoendoscope was developed (Figure 1) [14]. It enabled endoscopists to track a needle in real time across the image plane into a target lesion (Figure 2A). Furthermore, digital instruments also permitted the use of Doppler technology to assess vascular flow. Vilmann et al. created special needle equipment for biopsy using a linear-array echoendoscope, and reported the first case of endoscopic ultrasound-guided fine needle aspiration (EUS-FNA) of a pancreatic head lesion using a curved linear-array echoendoscope [15]. Endoscopic ultrasound-guided fine needle biopsy (EUS-FNB) was subsequently developed, which allows core samples to be collected by shearing tissue from the target lesion (Figure 2B) [16]. Cutting needles are expected to improve diagnostic accuracy, as well as provide tissue with conserved architecture, enabling histological analysis. The difference between EUS-FNA and EUS-FNB is basically the structure of the needles. Thus, they are both categorized as EUS-TA.

For pathological diagnosis of a solid pancreatic mass, EUS-TA has been proven to be highly accurate (sensitivity 85–89% and specificity 96–100%) by three meta-analyses [17,18,19]. A recent prospective study showed that the sensitivity of EUS-FNA for the diagnosis of a solid pancreatic mass is now over 90% [20,21]. EUS-TA is a safe, cost effective, and accurate technique [19], and has become the standard diagnostic test for PC. Recently, EUS-TA has also been used for examinations of gastrointestinal submucosal lesions [22,23], mediastinal and abdominal lymph nodes [24,25], and malignant biliary stricture [26].

The role of EUS-TA continues to expand. There are two main types of evolution regarding EUS-TA. One is interventional EUS [27,28,29,30,31,32,33,34,35,36,37,38,39,40,41,42,43,44,45]. Although EUS was initially designed as a tool for imaging the pancreatobiliary system, the application of this modality has expanded due to its high-resolution imaging capabilities. Furthermore, with subsequent advances in EUS devices, especially for echoendoscopes with larger instrument-channel diameters capable of allowing the insertion of stents, it offers alternative access routes from the gastrointestinal tract to surrounding structures. 

The other evolution is biomarker analysis using EUS-TA samples. Although EUS-TA has already been proven to be highly accurate, inconclusive results persist in cytopathology. Furthermore, predictive markers for therapeutic stratification of PC are still lacking. Thus, potentially promising biomarkers have been investigated. Advances in EUS-TA devices and molecular biology techniques permit the analysis of molecular biomarkers either on DNA, RNA, or microRNA using EUS-TA samples of PC.

## 3. Next-Generation Sequencing (NGS)

Precision medicine refers to the tailoring of treatment based on an individual’s genetics, lifestyle, and environment, and characterizing genomic aberrations in tumors for predictive and prognostic purposes by genome sequencing has become an integral part of precision medicine [46,47]. Currently, two methods, Sanger sequencing and NGS, are widely used in genome sequencing. The concept behind both sequencing techniques is similar in principle. In both sequencing techniques, DNA polymerase adds fluorescent nucleotides one by one onto a growing DNA template strand, and each incorporated nucleotide is identified by its fluorescent tag.

Sanger sequencing, which is relatively simple and easy to use, is the gold standard for DNA sequencing [48]. However, target DNA is copied only one fragment at a time, making this method costly, and time- and labor-intensive for large-scale sequencing. Furthermore, substantial amounts of DNA are required. NGS platforms sequence a massive parallel collection of clonally amplified or single DNA molecules that are spatially separated in a flow cell [49]. This feature facilitates the sequencing of millions to billions of short fragments of DNA [50,51]. This is an important advantage that enables the screening of large numbers of samples in a short period of time. Furthermore, NGS technology requires a relatively low amount of DNA or RNA in contrast to traditional sequencing technologies, and decreases the overall cost of multiple-marker screening [52]. Sanger sequencing was used in the sequencing of the first human genome that was completed in 2003 through the Human Genome Project, a 13 year effort with an estimated cost of $2.7 billion [53]. Today, sequencing the human genome using NGS technology only costs about $1000 and can be completed in just a few days.

Development of NGS technologies has increased the speed and reduced the cost of sequencing the nucleic acids of cancer cells. This can provide a comprehensive view of an individual patient’s cancer, which can impact real-time clinical decision-making. For PC patients, precision medicine has not been well established. However, NGS can unveil various potential predictive genomic biomarkers for possible development of new treatments and precision medicine. 

## 4. Genetic Markers in Pancreatic Cancer

There are four genes mutated at high frequency in pancreatic ductal carcinoma (PDAC): *KRAS*, *CDKN2A*, *TP53*, and *SMAD4*. These are referred to as driver genes [54]. Among these genes, *KRAS* mutation is the most studied oncogene [55,56,57,58,59,60,61,62,63]. *KRAS* mediates signaling the downstream signaling pathway from growth factor receptors such as epidermal growth factor receptor (EGFR). Mutation in the *KRAS* oncogene results in gain-of-function and activates the extracellular signal regulated kinase pathway (MAPK-ERK pathway) [64]. Once this pathway is activated, it translocates to the nucleus and promotes transcription activity for target genes that are involved in cell survival, growth, and proliferation [65]. The *KRAS* oncogene also interacts and activates other signaling molecules found to be related to stress response and cell growth such as JNK and PKC [66]. *KRAS* mutation has been found in over 95% of PDACs patients [67]. Furthermore, *KRAS* mutation is also detected in pre-cancerous lesion PDAC as intraepithelial neoplasias (PanIN) [54] and intraductal papillary mucinous neoplasm (IPMN) [68]. The other three genes were called tumor suppressor genes. *CDKN2A* is the most frequently altered tumor suppressor gene, which encodes an essential cell cycle regulator in more than 90% of PDAC [69]. Somatic mutations in the *TP53* tumor suppressor gene are frequently identified in 40–75% of PDACs [70]. Protein p53 encoded by *TP53* has a key role in the cellular stress response and cell cycle regulation [69]. The tumor suppressor gene *SMAD4* mediates signaling downstream of the TGFβ receptor and is inactivated in about 50% of PDACs [69]. Among of these gene mutations, alterations in both *KRAS* and *CDKN2A* have been detected at the early stage in pancreatic carcinogenesis. By contrast, *TP53* and *SMAD4* are mutated in a later stage [71,72].

## 5. Clinical Utility of EUS-TA for NGS

EUS-TA is an established technique for the diagnosis of pancreatic lesions with both high sensitivity and specificity. However, even though NGS can sequence multiple genes in limited samples [49,52,73], acquisition of a relatively large sample is mandatory for NGS. Pancreatic lesions are morphologically classified into solid pancreatic masses and pancreatic cystic lesions. NGS using EUS-TA samples has been also reported for solid pancreatic masses and pancreatic cystic lesions separately.

### 5.1. Solid Pancreatic Masses

Approximately 90% of PCs are PDACs, and mutational activation of the *KRAS* oncogene has been found in over 95% of PDAC patients [67]. Thus, NGS has been validated using *KRAS* mutation as a reference gene. Kameta et al. showed that NGS using EUS-TA samples was successfully established with high clinical sensitivity. In their analysis, 38 samples (27 PDACs, 11 non-PDACs including pancreatic neuroendocrine tumor (PanNET) and chronic pancreatitis) were analyzed with NGS using EUS-TA samples [74]. *KRAS* mutations were detected in 26 of 27 PDAC samples (96%) and none of the 11 non-PDAC samples (0%).

Molecular analysis could be improved by NGS even using EUS-TA samples. De Biase et al. analyzed *KRAS* mutations of 60 samples obtained by EUS-FNA, and compared three different techniques: Sanger sequencing, NGS, and allele specific locked nucleic acid quantitative PCR [75]. In their study, the sensitivity for detecting the *KRAS* mutation was 42.1% for Sanger sequencing, 73.7% for NGS, and 52.8% for allele specific locked nucleic acid quantitative PCR. They showed that NGS increased the clinical sensitivity without decreasing the specificity.

Several studies have examined the surrogacy of EUS-TA samples for surgically resected specimens in NGS. The results of these studies of NGS using EUS-TA samples showed high concordance with that in a surgically resected specimen used as a reference standard. These findings are potentially important because most patients with PC are ineligible for surgical resection. At present, surgical resection is the only potential curative treatment, and precision medicine is expected to substantially benefit these patients. Valero et al. performed preoperative EUS-FNA and simulated FNA in intra-operative frozen sections using a 19-gauge needle [76]. Comparison of both results of NGS revealed a concordance frequency of 100% for all driver genes present. Gleeson et al. assessed multigene mutational concordance between EUS-FNA samples and surgically resected specimens using an NGS panel of 160 genes in pancreatic and ampullary cancer and found an absolute concordance in 83% of the cases [77].

No prospective trials have evaluated the adequacy of EUS-TA samples for NGS, but several retrospective studies have reported that the adequacy of EUS-TA samples for NGS ranged from 60% to 100% [78,79,80,81] (Table 1).

Young et al. performed NGS using formalin fixed paraffin embedded (FFPE) samples obtained by EUS-FNA. Using a customized gene panel (287 genes), genomic profiles were generated successfully from 23 of 23 (100%) solid pancreatic masses, and the most common mutations were observed in *KRAS* (78%), *TP53* (74%), *CDKN2A/B* (35%), *SMAD4* (17%), and *PTEN* (13%) [80].

Elhanafi evaluated the adequacy of EUS-TA samples for NGS in PDAC [79]. Of a total of 167 samples (145 via EUS-FNA, 22 via EUS-FNB), the adequacy rate for NGS was 70.1%. EUS-FNB resulted in a higher proportion of patients with sufficient sample for NGS compared with EUS-FNA (90.9% vs. 66.9%, respectively; *P* = 0.02)

Larson et al. evaluated the adequacy of biopsy samples for NGS in 76 patients with pancreatic exocrine malignancy (74 with PDAC) [78]. The volume of tissue obtained ranged from 0.008 cm^3^ to 0.36 cm^3^ (median, 0.072 cm^3^). Of a total of 76 samples, 61 samples were obtained via EUS-TA (7 via EUS-FNA, 54 via EUS-FNB). They reported the adequacy rate of EUS-TA for NGS was 67.2% (EUS-FNA, 42.9%; EUS-FNB, 70.4%).

Gleeson et al. assessed the results of NGS for PanNET using a customized panel of 15 genes [81]. Their selection criteria were: at least 3000 total nucleated cells and ≥20% tumor cells in a background of benign nucleated cells in cytology single slide smear specimens. In their retrospective study, a total of 156 samples were obtained from primary pancreatic lesions via EUS-FNA cytology smear, with an adequacy rate of 58%.

In solid-pseudopapillary neoplasms (SPNs), neoplastic cells harbor somatic point mutations in exon 3 of *CTNNB1*. *CTNNB1* mutation is considered a unique genetic characteristic of SPNs [82]. Kubota et al. analyzed *CTNNB1* mutation by NGS in 38 samples (7 SPNs, 16 PDACs, and 11 PanNETs) obtained by a 22-gauge EUS-FNA. All SPN samples showed *CTNNB1* mutation in their study. In contrast, *CTNNB1* mutation was not observed in any other pancreatic disease, except for a single case of PanNET. Springer et al. also reported that *CTNNB1* mutations were identified by NGS in all SPN samples in their retrospective study [83].

### 5.2. Pancreatic Cystic Lesions

Pancreatic cysts are not uncommon findings on abdominal imaging, with the reported incidence ranging from 2.6% to 13.5% in asymptomatic patients and increasing in prevalence with older age [84,85,86]. The clinical management of patients with pancreatic cysts is mainly based on clinical presentation and cyst fluid analysis [87]. However, management of pancreatic cysts is still challenging. Pergolini et al. reported in a large retrospective analysis that approximately 70% of resected IPMNs harbor only low-grade dysplasia, and these also could have been safely observed [88]. Consequently, EUS-TA is expected to play a complementary role in the clinical management of pancreatic cysts. However, the accuracy of pathological examination by EUS-TA is limited compared to that for solid pancreatic masses. A meta-analysis comprising 18 studies and 1438 patients showed that EUS-FNA was moderately accurate (pooled sensitivity and specificity were 54% and 94%, respectively) [89]. Another meta-analysis also showed good specificity but poor sensitivity for cytology in differentiating benign from malignant IPMN vis EUS-FNA (pooled sensitivity and specificity were 64.8% and 90.6%, respectively) [90]. Thus, additional ancillary studies are needed for the diagnosis of pancreatic cystic lesions.

Recently, the utility of molecular analysis of pancreatic cyst fluid samples has been reported. Mutations in *KRAS* are commonly detected in IPMNs and mucinous cystic neoplasms (MCN)s and the presence of *GNAS* mutations is highly specific for IPMNs [91,92,93]. In contrast, genetic alteration in *VHL* are highly specific for serous cystadenomas (SCAs), and *CTNNB1* mutations in the absence of other genetic alterations are observed in SPNs [94]. In pancreatic cystic lesions, NGS is performed on cyst fluid samples containing cellular DNA and/or free DNA. Although few reports of NGS using EUS-TA samples from pancreatic cystic lesion are found, the adequacy rate of has been reported to be over 90% (Table 2). NGS using EUS-TA samples from pancreatic cystic lesions is potentially useful in clinical management of them. Jones et al. assessed NGS via EUS-FNA on the clinical diagnosis of pancreatic cysts using a custom panel of 39 cancer genes [95]. Of a total of 99 cystic lesions, NGS was successively performed in 96 lesions (97%), and the median DNA content for NGS was 12.3 ng (range 0–1283 ng). Based on the 18 patients with pathological confirmation of disease, NGS showed a sensitivity of 86% and a specificity of 75% for detection of mucinous neoplasia.

Shinghi et al. reported the results of a large prospective study evaluating preoperative NGS for pancreatic cystic lesions [96]. In their study, 626 of 673 samples (93%) of pancreatic cystic fluid obtained via EUS-FNA were satisfactory for NGS. *KRAS/GNAS* mutations by NGS were associated with 89% sensitivity and 100% specificity for a mucinous neoplasia. In comparison, *KRAS/GNAS* mutations by Sanger sequencing had 65% sensitivity and 100% specificity. Furthermore, for NGS, the combination of *KRAS/GNAS* mutations and *TP53/PIK3CA/PTEN* alterations increased sensitivity and specificity up to 89 and 100% for advanced neoplasia, respectively. In contrast, ductal dilatation, mural nodule, and malignant cytopathology had lower sensitivities (42%, 32%, and 32%, respectively). They concluded that preoperative NGS for pancreatic cystic lesions for *KRAS/GNAS* mutations is highly sensitive for mucinous neoplasms compared with Sanger sequencing. Moreover, the combination of *TP53/PIK3CA/PTEN* alterations was a useful preoperative marker for advanced neoplasia.

Springer et al. reported the result of a multi-center retrospective study of 130 patients with resected pancreatic cystic neoplasms (96 IPMNs, 12 SCAs, 12 MCNs, and 10 SPNs) [83]. In their study, the DNA concentration obtained from pancreatic cyst fluid collected by EUS-FNA or resected specimens was 4.9 ng/μL (range, 0.05–270 ng/μL). There were distinct mutational profiles associated with each type of cyst: *KRAS* mutation in 78% and *GNAS* mutation in 58% of IPMNs, *VHL* mutation in 42% of SCAs, and *KRAS* mutation in 50% of MCNs. In total, NGS correctly identified 67 of the 74 (91%) patients who did not require surgery, suggesting it may be useful for avoiding unnecessary surgical resection.

Recently, novel through-the-needle biopsy forceps for EUS-TA (Moray micro forceps, US Endoscopy, Mentor, OH, USA) have been developed. The device easily passes through the 19-gauge EUS-TA needle and allows tissue samples from the pancreatic cyst wall to be collected. Zhang ML et al. reported the result of a retrospective study comparing through-the-needle biopsy forceps and pancreatic cyst fluid analysis for diagnosis of pancreatic cystic lesions. In this study, the diagnostic performance of through-the-needle biopsy forceps was comparable with pancreatic cyst fluid analysis (58.3 vs. 60.4%; *P* = 0.949, respectively). However, through-the-needle biopsy forceps were found to be superior for diagnosing specific cyst subtypes compared with pancreatic cyst fluid analysis (50.0 vs. 18.8%; *P* < 0.001, respectively) [97]. Shakhatreh et al. also reported small case series about the utility of through-the-needle biopsy forceps for pancreatic cysts [98]. The through-the-needle biopsy forceps are expected to improve diagnostic performance of pancreatic cysts.

## 6. MicroRNAs (miRNA)

MicroRNAs (miRNAs), are small (19–25 nucleotides), single-stranded RNA molecules that regulate gene expression [99]. miRNAs are highly stable in tissues and fluids, and they can be quantified in very low amounts of material and in highly degraded samples. Various studies have shown the differential expression of miRNAs between normal and malignant tissues, and their association with cancer development, diagnosis, and assessment of prognosis [100]. MicroRNAs have also been shown to directly function as oncogenes or tumor suppressors [101,102,103].

Aberrant expression of several miRNAs has been detected in PC [104,105,106,107], and their application as potential biomarkers in clinical samples are expected. Several studies showed the utility of NGS of miRNAs from pancreatic cyst fluid [108,109]. Matthaei et al. extracted miRNAs from microdissected FFPE specimens or fluid specimens from IPMNs, and assessed their diagnostic utility. They reported that a predictive model using nine miRNAs had 89% sensitivity and 100% specificity for differentiation of cystic lesions that required surgical resection (high-grade IPMN, PanNET, and SPN) [108]. Wang et al. reported that NGS analysis revealed 15 miRNAs in cystic fluid samples from invasive IPMN. The miRNAs were identified from 18 to 40 nucleotides long small RNA derived from bar-coded libraries yielding 10–15 million reads from each sample. This amount of read data is expected to detect even transcripts with low expression levels, thus allowing robust identification of differentially abundant miRNAs, irrespective of their expression levels among the samples analyzed [109].

For application of miRNA analysis using EUS-TA samples, further examination is needed. However, miRNA may prove essential for helping clinicians diagnose and treat PC.

## 7. How to Obtain Adequate Samples for NGS via EUS-TA?

Cellularity and tumor fraction are important for NGS. Samples with low cellularity have an increased risk of insufficient FNA and polymerase chain reaction failure, whereas samples with low tumor fractions have an increased risk of false-negative results [110]. Although tissue samples obtained by EUS-TA provide high diagnostic accuracy, the total amount of obtained sample is limited. While NGS allows sequencing of multiple genes in limited samples [49,52,73], the acquisition of relatively large tumor samples to avoid contamination is mandatory for NGS.

### 7.1. Target Site

**Figure 3:**
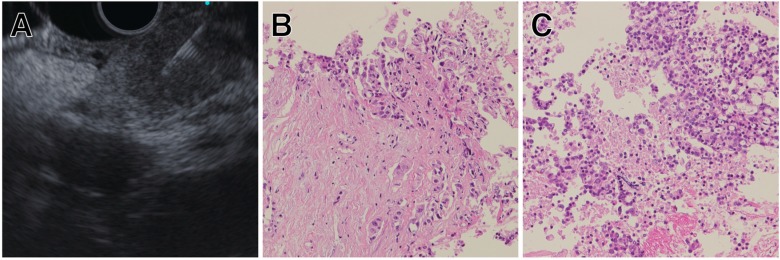
Representative case of EUS-TA for PC. (**A**) EUS-FNB of PC located in the tail of the pancreas. (**B**) Tissue sample from primary tumor containing more stromal cells than neoplastic cells. (**C**) Tissue sample from lymph node metastasis showing higher tumor fraction. This sample ware utilized for next-generation sequencing (NGS).

Generally, PC tissue contains stromal cells or hematopoietic cells, and may even contain more desmoplastic fibroblasts than tumor cells [111]. Therefore, the use of NGS is difficult due to possible contamination by desmoplastic fibroblasts. In contrast, Torphy et al. reported that solid organ metastasis (liver and lung) and lymph node metastasis from PC had lower desmoplastic stroma density compared with primary tumor [112]. A recent retrospective study showed the diagnostic accuracy of EUS-FNA combined with contrast-enhanced harmonic EUS for hepatic lesions was 86.7%, and there were no procedure-related adverse events. Concurrent EUS-FNA both for pancreatic and liver lesions was performed in 18/30 patients (60%) in this study [113]. These findings suggested that EUS-TA from liver metastasis or lymph node metastasis could be a complementary approach when the sample obtained from primary tumor is insufficient for NGS (Figure 3A–C).

### 7.2. Rapid On-Site Evaluation (ROSE)

Obtaining an adequate sample is fundamental to making an accurate diagnosis with EUS-TA and requires a team effort between the endosonographer and cytopathologist. The aim of Rapid On-Site Evaluation (ROSE) is to provide real-time feedback about the content and adequacy of a specimen in order to make the most accurate diagnosis, with the minimum number of passes, thus maximizing the efficiency of the procedure. It has been reported that the presence of an on-site cytopathologist led to a 10–15% increase in diagnostic yield [114,115]. However, the benefit of ROSE may be limited in EUS-TA for NGS. Recent data suggested that ROSE may have a role during the learning phase of EUS-FNA only, and in centers where the specimen adequacy rate is low (<90%) [116]. The direct benefit of ROSE may be limited to the decrease in the number of unsatisfactory samples, thereby reducing the need for more passes, and this may potentially reduce patient risk.

Another potential advantage of ROSE is high-quality specimen preparation and the adequate triage of limited specimens [117]. If the clinician can utilize cytological smears or FNA rinse samples for NGS, the best scenario is that one more pass for sampling for NGS will be made after feedback regarding the adequacy of the specimens by ROSE.

### 7.3. Which Should We Choose, EUS-FNA or EUS-FNB?

The most important issue regarding sampling for NGS may be selection between EUS-FNB and EUS-FNA. However, the available literature comparing EUS-FNA and EUS-FNB does not provide definitive results.

**Figure 4:**
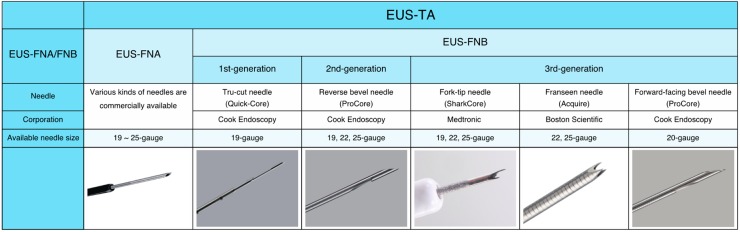
Differences in needle design between EUS-FNA and EUS-FNB needles.

EUS-FNA has become an increasingly important tool to achieve a definitive diagnosis with high accuracy for pancreatic lesions. However, diagnostic failures are usually caused by various factors, including inadequate samples and necrotic or fibrotic tumors in which viable cells are difficult to obtain [118]. To overcome these limitations of EUS-FNA, EUS-FNB needles have been developed to preserve the tissue architecture and improve the sample adequacy and diagnostic accuracy (Figure 4). An EUS compatible Tru-cut biopsy needle (Quick-Core, Cook Endoscopy, Limerick, Ireland) was initially developed, but the inherent stiffness of the 19-gauge needle and difficulty with the firing mechanism made this device difficult to use, particularly for sampling the uncinate process of the pancreas. Consequently, a second-generation EUS-FNB needle, the Reverse bevel needle (ProCore, Cook Endoscopy, Limerick, Ireland) with a side opening and a reverse bevel, was developed. This second-generation EUS-FNB needle theoretically improves the diagnostic yield and is optimal for NGS. However, the superiority of the diagnostic accuracy and the sample adequacy of the second-generation EUS-FNB needle over the conventional EUS-FNA needle remain controversial. In a recent randomized, controlled trial by Cheng et al., the diagnostic yield of the second-generation EUS-FNB needle was significantly higher compared with the conventional EUS-FNA needle (91.44% vs. 80.0%, respectively; *P* = 0.0015) [119]. However, there was no significant difference between the second-generation EUS-FNB needle and the conventional EUS-FNA needle in four randomized, controlled trials [120,121,122,123] and a meta-analysis [124].

Recently, third-generation EUS-FNB needles, a Fork-tip needle with two opposite cutting edges (SharkCore, Medtronic Corporation, Newton, MA), a Franseen needle with geometric tip having three incorporated cutting edges (Acquire, Boston Scientific Corporation, Natick, MA), and a forward-facing bevel needle (20-gauge ProCore, Cook Endoscopy, Limerick, Ireland) with a Menghini tip have been developed.

To date, there has been only one randomized trial comparing the third-generation EUS-FNB needles and the conventional EUS-FNA needle. van Riet et al. reported the results of a multi-center randomized trial of 608 patients with a solid lesion (312 pancreatic lesions, 147 lymph nodes, and 149 other lesions). In this trial, the diagnostic accuracy of third-generation EUS-FNB needles (forward-facing bevel needle) showed superiority both in accuracy and tissue core procurement compared with the 25-gauge EUS-FNA needle (accuracy: 87 vs. 78%; *P* = 0.002, tissue core procurement: 77 vs. 44%; *P* < 0.001, respectively) [21]. In a retrospective case control study, the third-generation EUS-FNB needle (Fork-tip needle) achieved higher histological yield compared with the conventional EUS-FNA needle (95 vs. 59%, respectively; *P* = 0.01) [125]. Armellini et al. also reported the results of large retrospective trial. In this trial, the third-generation EUS-FNB needle (forward-facing bevel needle) showed superiority in tissue core procurement compared with the conventional EUS-FNA needle (92.6 vs. 49.5%, respectively; *P* < 0.0001) [126]. In another large retrospective study, the frequency of obtaining adequate samples for cytology using the third-generation EUS-FNB needle (Fork-tip needle) was comparable with the conventional EUS-FNA needle (94.1% vs. 92.7%, respectively). However, the median number of passes to obtain a tissue diagnosis using the third-generation EUS-FNB needle (Fork-tip needle) was significantly less compared with the conventional EUS-FNA needle (one vs. three, respectively; *P* < 0.001) [127].

In a comparison between second- and third-generation EUS-FNB needles, the third-generation EUS-FNB needle (Fork-tip needle) showed superiority both in sensitivity and accuracy compared with the second-generation EUS-FNB needle (sensitivity: 90.1 vs. 71.1%; *P* = 0.006, accuracy: 92 vs. 74%; *P* = 0.0006, respectively). In this prospective cohort study, the proportion of samples classified as adequate for histological analysis was also significantly higher for the third-generation EUS-FNB needle (Fork-tip needle) compared with the second-generation EUS-FNB needle (99 vs. 87%, respectively; *P* = 0.002) [128].

Among the third-generation EUS-FNB needles, a randomized, controlled trial showed that there is no significant difference in the yield of histological tissue between the Franseen needle and the Fork-tip needle (96.0 vs. 92.0%, respectively; *P* = 0.32) [129].

Although strong evidence based on randomized, controlled trials is insufficient, these results potentially indicate that the third-generation EUS-FNB needles allow higher histological yield compared with both the conventional EUS-FNA needle and the second-generation EUS-FNB needle.

NGS commonly requires a tumor fraction ≥20% [130,131]. Newer EUS-FNB needles may enable acquisition of adequate core tissue samples, and these samples can be processed into FFPE specimens (cell blocks). FFPE specimens have an advantage of direct tumor cellularity evaluation. Furthermore, FFPE samples preserve the tissue architecture and can be used for future ancillary studies. Thus, FFPE specimens were once preferred over cytological smear specimens [132]. In a retrospective study, Larson et al. reported that EUS-FNB was not significant, but tended to be highly correlated with adequacy compared with EUS-FNA (70.4 vs. 42.9%, respectively: *P* = 0.1494) [78]. Elhanafi et al. reported EUS-FNB is more likely to result in sufficient tissue sampling for NGS compared with EUS-FNA (90.9 vs. 66.9%, respectively; *P* = 0.02). After adjustment, EUS-FNB was an independent predictive factor of sample adequacy for NGS (adjusted odds ratio 4.95, 95% confidence interval 1.11–22.05). EUS-FNB was more likely to obtain an adequate tissue sample for NGS from tumors ≤3 cm (100 vs. 68.4%, respectively: *P* = 0.017) and tumors located in the pancreatic head/neck (100 vs. 63.1%, respectively: *P* = 0.03) [79].

However, the excellent performance of cytological smear specimens in molecular analyses has been shown recently [133]. The quality of DNA in FFPE samples is compromised due to the formalin-fixation and processing, since FFPE samples degrade DNA with consequent production of artificial mutations and decreased bioavailability [134]. It is not uncommon to encounter insufficient cellular cell block, and this can contribute to inadequate DNA for NGS. Hartley et al. compared 30 matched EUS-FNA cytological smears and macrodissected FFPE samples obtained by surgical resection. In their study, DNA yield per nuclear area was higher in EUS-FNA cytological smears compared with FFPE samples (0.86 ng/mL vs. 0.51 ng/mL, respectively: *P* = 0.0051). They concluded that FNA samples are a more optimal source of DNA, and represent an important resource for molecular analysis [135].

Several studies have reported the excellent performance of FNA rinse samples in molecular analyses. EUS-TA samples are routinely rinsed and fixed in an alcohol-based fixative immediately after their acquisition, and this contributes to excellent preservation and quality of DNA and RNA. Wei et al. compared the performance of FNA rinse samples and that of cell block samples used for NGS [136]. In their study, the tumor fraction in the FNA rinse samples ranged from 10 to 80%. NGS was successfully performed using all samples. Much more DNA was obtained from the FNA rinse samples compared with the paired cell block samples (176.3 vs. 10.6 ng/µL, respectively). They also reported perfect concordance of the results of NGS between the liquid cytology samples, including FNA rinse material and the surgically resected specimen.

In lung cancer, the College of American Pathologists guidelines state that pathologists may use either FFPE samples (cell blocks) or other cytological preparations (cytological smears) as suitable specimens for biomarker molecular testing, including NGS [137]. However, in most situations, NGS is performed using FFPE samples. Future research should focus on whether EUS-FNA samples or EUS-FNB samples are better for performing NGS.

### 7.4. Needle Size: Small versus Large

At present, various different sizes of needles for EUS-TA are available: 19- to 25-gauge needles. Theoretically, larger needles can obtain a larger amount of material. However, the largest 19-gauge needle tends to be stiffer and may be of limited value in situations where the endoscope needs to be flexed (i.e., uncinate process of the pancreas). In a randomized study comparing 19-gauge nitinol needle and conventional 22-gauge needle for transduodenal EUS-TA, the diagnostic accuracy of the 19-gauge nitinol needle was inferior to the 22-gauge needle (69.5 vs. 87.3%; *P* = 0.02, respectively). In this study, cytological and histological qualities were not statistically different between the 19-gauge nitinol needle and 22-gauge needle (cytological quality score: 6.9 vs. 8.6; P = 0.09, histological quality score: 8.0 vs. 8.6; *P* = 0.27, respectively) [138]. In contrast, passage of the thinner 25-gauge needle may be easier when the endoscope is angulated, as in transduodenal access to a pancreatic head lesion. Thus, clinicians tend to prefer 22-gauge and 25-gauge needles.

Randomized controlled studies suggest that there is no incremental diagnostic yield with 19-gauge needles compared with conventional 22-gauge needles [139,140]. Conversely, two meta-analyses have shown slight superiority of the thinner 25-gauge needle over the conventional 22-gauge needle for solid pancreatic masses [141,142]. The reason for the observed superior diagnostic accuracy of thinner needles in these analyses remains uncertain. One possibility is that thinner needles are associated with fewer bloody aspirates than larger needles [143], and this may potentially have a beneficial effect on cytological interpretation without compromising cellular yield.

However, a recent randomized trial by van Riet et al. reported that a third-generation 20-gauge EUS-FNB needle with high flexibility (forward-facing bevel needle) showed superiority in tissue core procurement compared with the 25-gauge EUS-FNA needle (77 vs. 44%; *P* < 0.001, respectively) [21]. In another retrospective study, Larson et al. also reported that larger needle size was highly correlated with adequacy for EUS-FNB (19-gauge, 100%; 22-gauge, 78%; and 25-gauge, 0%) [78], and they concluded that all biopsies including EUS-FNB were more likely to be successful using larger-gauge needles. Newly developed needles and techniques may minimize blood contamination and enable high-quality specimens to be obtained. Considering the amount of sample obtained and needle flexibility, a 22-gauge needle (or third-generation 20-gauge EUS-FNB needle) may be suitable for NGS.

### 7.5. Technique: Suction vs, Non-Suction (Slow-Pull Technique)

In the conventional EUS-TA procedure, after needle puncture of the target lesion, the typical technique includes stylet removal followed by to-and-fro movements with negative suction. However, application of suction during EUS-TA may damage the cellular structure and result in blood contamination while obtaining samples [144,145]. In contrast to conventional suction techniques using a syringe, the slow-pull technique, which was recently introduced for EUS-TA, minimizes negative pressure by removing the stylet itself from the needle slowly and continuously. This may enable the acquisition of high-quality specimens with minimal blood contamination [146,147,148].

Lee et al. compared three methods for EUS-TA without ROSE. The slow-pull technique (A), conventional negative suction technique after stylet removal (B), and non-suction technique after stylet removal (C). Although the rate of adequate core tissue acquisition was not statistically different, the rate of a good or excellent proportion of cellularity was highest in group A, and blood contamination was more prevalent in group B [149]. These results indicate that the slow-pull technique may provide more adequate samples for NGS, i.e., less blood contaminated and a sample with higher tumor fraction.

## 8. Conclusions

Newer EUS-FNB needles have achieved both higher diagnostic rates and good histological retrieval rates. Consequently, EUS-TA has been developed to acquire tissue samples that facilitate the ability to perform NGS. This advance opens the way to target therapies for PC. On the other hand, the adequacy of EUS-TA samples for NGS ranges widely, from 60 to 100%. Furthermore, it remains uncertain whether EUS-FNB needles are better than EUS-FNA needles in the setting of NGS [150]. Thus, prospective trials are necessary to establish the utility of NGS using EUS-TA samples.

## Figures and Tables

**Table 1:** Adequacy rate of EUS-TA samples for NGS in pancreatic solid mass.

Author (Year)	Study Type	No. of Patients	Type of Tumor	Biopsy Type	Needle	Adequacy Rate for NGS	*P*-Value	Required Tumor Fraction	Genes Targeted	Frequency of Genomic Alterations
Elhanafi S, et al. (2018)	Retrospective cohort study	167	PDAC	EUS-FNA/B		70.1%		≥10%	Custom panel (47 genes)	*KRAS* (88%), *TP53* (68%)*, SMAD4* (16%)
		145		EUS-FNA	EUSN-3 (22-gauge)	66.9%	0.02			
		22		EUS-FNB	SharkCore/ProCore (22-gauge)	90.9%				
Larson BK, et al. (2018)	Retrospective study	61	Pancreatic exocrine malignancy	EUS-FNA/B		67.2%		≥20%	FoundationOne (315 genes)	NA
		7		EUS-FNA	NA	42.9%	0.1494			
		54		EUS-FNB	SharkCore/ProCore	70.4%				
Gleeson FC, et al. (2017)	Retrospective study	156	PanNET	EUS-FNA	NA	58%		≥20%	Custom GeneRead DNAseq Targeted Panel V2 (15 genes)	*MEN1* (42%), *DAXX* (11%), *ATRX* (10%), *TSC2* (8%)
Young G, et al. (2013)	Retrospective study	23	PDAC, Mucinous adenocarcinoma, adenocarcinoma NOS, PanNET	EUS-FNA	NA	100%		≥20%	Custom panel (287 genes)	*KRAS* (78%), *TP53* (74%)*, CDKN2A/B* (35%)*, SMAD4* (17%)*, PTEN* (13%)
Gleeson FC, et al. (2016)	Retrospective study	47	PDAC, Ampullary adenocarcinoma, IPMN, Lynch syndrome associated PDAC	EUS-FNA	NA	61.7%		≥20%	Human Comprehensive Cancer GeneRead DNAseq Targeted Panel V2 (160 genes)	*KRAS* (93.1%), *TP53* (72.4%)*, SMAD4* (31%), GNAS (10.3%)

NGS, next generation sequencing; PDAC, pancreatic ductal adenocarcinoma; NOS, not otherwise specified; PanNET, pancreatic neuroendocrine tumor; IPMN, intraductal papillary mucinous neoplasm; EUS-FNA, endoscopic ultrasound-guided fine needle aspiration; EUS-FNB, endoscopic ultrasound-guided fine needle biopsy; NA, not available.

**Table 2:** Summary of studies evaluating NGS using EUS-TA samples in pancreatic cystic lesion.

Author (Year)	Study Type	No. of Patients	Type of Lesion	Biopsy Type	Needle	Adequacy Rate for NGS	Genes Targeted	Genomic Alteration Detected	Frequency of Genomic Alterations
Singhi AD, et al. (2018)	Prospective study	673	IPMN, MCN, SCA, Cystic PanNET, Acinar cell cystadenoma, Pseudocyst	EUS-FNA	NA	93%	PancreaSeq (10 genes)	57%	*KRAS* (42%), *GNAS* (26%)*, BRAF* (1%)*, SMAD4* (17%)*, CTNNB1* (1%)
Jones M, et al. (2016)	Prospective study	99	IPMN, MCN, SCA, Cystic PanNET, NOS	EUS-FNA	NA	97%	Custom panel (39 genes)	57%	*KRAS* (47%), *GNAS* (24%)
Springer S, et al.	Retrospective study	24	17 IPMN, 3 MCN, 2 SCA, 1 SPN, 1 ITPN	EUS-FNA	NA	NA	Custom panel (11 genes)	87.5%	NA

NGS, next generation sequencing; IPMN, intraductal papillary mucinous neoplasm; MCN, mucinous cystic neoplasm; SCA, serous cystadenoma; PanNET, pancreatic neuroendocrine tumor; NOS, not otherwise specified; SPN, solid-pseudopapillary neoplasm; TIPN, intraductal tubulopapillary neoplasm; EUS-FNA, endoscopic ultrasound-guided fine needle aspiration; NA, not available.
